# Statistics of Medical Schools

**Published:** 1851-05

**Authors:** 


### ARTICLE III.

### STATISTICS OF MEDICAL SCHOOLS—SESSION 1850—51.

| | Students. | Graduates. |
|---|---|---|
| Jefferson Medical College, | 504 | 227 |
| University of Pennsylvania, | 466 | 167 |
| University of the City of New York, | 411 | 116 |
| College of Physicians and Surgeons, N. Y., | 230 | 60 |
| Cleveland Medical College, | 202 | |
| Philadelphia College of Medicine, (2 sessions) | 244 | 72 |
| Medical College of Georgia, | 159 | 50 |
| Ohio Medical College, | 186 | 59 |
| Buffalo Medical College, | 115 | 30 |
| Rush Medical College, | 132 | 30 |
| Geneva Medical College, | 101 | |
| Starling Medical College, | 125 | 35 |
| Castleton Medical College, (2 sessions,) | 153 | 64 |
| Pennsylvania Medical College, | | 36 |
| University of Maryland, | | 45 |
| University of Louisiana, | | 37 |
| Albany Medical College, | 93 | 24 |
| New York Medical College, | 40 | 12 |
| Washington University of Baltimore, | | 13 |

| | Students. | Graduates. |
|---|---|---|
| Kentucky School of Medicine, | 100 | |
| University of Virginia, | 381 | 24 |
| Indiana Central Medical College, | | 18 |
| Keokuk (late Rock Island,) School, | | 10 |
| Dartmouth Medical College, | | 9 |
| Yale College, | | 11 |
| Harvard University, | | 10 |
| University of Missouri, | | 83 |
| Baltimore College of Dental Surgery, | | 17 |
| Philadelphia College of Pharmacy, | 82 | 19 |

